# Investigating the crosstalk between *ABCC4* and *ABCC5* in 3T3-L1 adipocyte differentiation

**DOI:** 10.3389/fmolb.2024.1498946

**Published:** 2024-12-09

**Authors:** Ankit P. Laddha, Aniket Wahane, Raman Bahal, José E. Manautou

**Affiliations:** Department of Pharmaceutical Sciences, University of Connecticut, Storrs, CT, United States

**Keywords:** 3T3-L1 cells, ABCC transporter, adipogenesis, lipids, siRNA, cAMP

## Abstract

**Introduction:**

The plasma membrane-bound protein, multi-drug resistance-associated protein 4 (*MRP4/ABCC4*), has gained attention for its pivotal role in facilitating the efflux of a wide range of endogenous and xenobiotic molecules. Its significance in adipogenesis and fatty acid metabolism has been brought to light by recent studies. Notably, research on *ABCC4* knockout (*ABCC4* ^*−/−*^) mice has established a link between the absence of *ABCC4* and the development of obesity and diabetes. Nevertheless, the specific contribution of *ABCC4* within adipose tissue remains largely unexplored.

**Methods:**

To address this gap, we conducted a study to elucidate the role of the *ABCC4* transporter in mature adipocytes, using siRNA constructs to silence its gene function.

**Results:**

The successful knockdown of *ABCC4* significantly altered lipid status and adipogenic gene expression in mature 3T3-L1 adipocytes. Intriguingly, this knockdown also altered the gene expression patterns of other *ABCC* transporter family members in 3T3-L1 cells. The downregulation of *ABCC5* expression was particularly noteworthy, suggesting potential crosstalk between *ABCC* transporters in mature adipocytes. Additionally, knocking down *ABCC5* resulted in significantly higher adipogenic and lipogenic gene expression levels. Oil Red O staining confirmed increased lipid accumulation following the knockdown of *ABCC4* and *ABCC5*. Surprisingly, the simultaneous knockdown of both transporters did not show a cumulative effect on adipogenesis, rather it led to higher levels of intracellular cAMP and extracellular prostaglandin metabolite, both of which are essential signaling molecules in adipogenesis.

**Conclusion:**

These results highlight the complex interplay between *ABCC4* and *ABCC5* transporters in adipocyte function and suggest their individual contributions toward obesity and related disorders.

## 1 Introduction

Obesity and diabetes have become increasingly prevalent worldwide over the past 3 decades (Tiwari and Balasundaram, 2023). Since 1975, global obesity rates have nearly tripled, presenting a significant risk factor for cardiovascular disease due to increased insulin resistance and inflammation (Bhupathiraju and Hu, 2016; Ortega et al., 2016). Overall, obesity has garnered considerable attention as a significant health hazard, underscoring the urgent need to identify novel factors involved in its progression. Our laboratory has recently made important contributions in identifying a novel genetic factor associated with the development of obesity and establishing its plausible molecular mechanism(s) (Donepudi et al., 2021).

Obesity is characterized by increased adipose tissue mass that results from hypertrophy and hyperplasia of adipocytes (Hajer et al., 2008). Adipocytes are specialized cells that play an important role in energy homeostasis (Luo and Liu, 2016). *ABCC4*, an ATP-binding cassette class of plasma membrane efflux transporter, has been reported to play an important role in adipose tissue physiology by regulating adipogenesis. Our laboratory has established that *ABCC4* knockout mice exhibited a noteworthy increase in adipose tissue weight and adipocyte hypertrophy compared to wild-type mice (Donepudi et al., 2021). Blocking *ABCC4* function in mice embryonic fibroblasts or pre-adipocyte cells using pharmacological inhibitors promotes adipogenesis by upregulating various signaling molecules involved in this process. However, there are concerns regarding the substrate selectivity and specificity of pharmacological inhibitors of *ABCC4*. MK-571, developed initially as a cysteinyl leukotriene receptor 1 antagonist, is a broad-spectrum *ABCC* inhibitor and thus does not display selectivity towards *ABCC4* (Bertollotto et al., 2018)*.* Moreover, there have been reports that other *ABCC* transporters, such as *ABCC1* and *ABCC5,* which transport similar substrates as *ABCC4,* also play an important role in adipogenesis (Cheung et al., 2014).

To investigate the specific role of *ABCC4* in adipose tissue physiology, we employed a loss of gene function approach utilizing siRNA. Our study utilized 3T3-L1 cells, derived from mouse embryonic fibroblasts, as a model system. Prior studies have reported difficulties in silencing genes using siRNA in differentiated 3T3-L1 cells with a standard lipid-based transfection system (Kilroy et al., 2009). In addition, the timing of siRNA treatment in 3T3-L1 cells impacts the degree of adipogenesis, as these cells require 8–10 days to differentiate fully.

To address these challenges, we have used the non-liposomal polymeric system TransIT-TKO (MirusBio, Madison, WI) and multiple siRNA treatments during the differentiation of 3T3-L1 cells. Following *ABCC4* silencing, we examined the compensatory regulation of other *ABCC* family transporters and their involvement in adipogenesis. This study highlights the selectivity of *ABCC4* silencing and its impact on the broader transport network within adipocytes.

## 2 Materials and methods

### 2.1 3T3-L1 cell differentiation

Murine 3T3-L1 cells (2 × 10^5^ cells) were cultured in 12 well plates using Dulbecco’s Modified Eagle’s Medium (DMEM) with high glucose (Gibco). The media was supplemented with 10% Fetal Bovine Serum, Qualified (Gibco), and 1% penicillin and streptomycin (Gibco). The cells were grown in complete media until they reached 100% confluency. Differentiation was initiated 2 days post-confluency using a MDI cocktail containing 0.5 mM isobutyl methylxanthine (IBMX), 1 μM dexamethasone, and 10 μg/mL insulin. After 48 h of induction, the media was changed to post-differentiation media (complete growth media with 10 μg/mL insulin). After an additional 48 h, the medium was again changed to complete growth media. The cells were maintained in complete media for 72–96 h. Eight days after adding the differentiation media, cells were fully matured to differentiated adipocytes.

### 2.2 Quantitative RT-qPCR

Total RNA was purified from the cultured cells using the phenol-chloroform standard isolation method. The total RNA was measured using NANODROP 2000c Spectrophotometer (Thermo Fisher Scientific). Complementary DNA was made using the High-Capacity cDNA Reverse Transcription Kit (Applied Biosystems, 4368814) according to the manufacturer’s protocol. Real-time PCR was performed using Universal Master Mix II, no UNG. (Catalog: 4440040) with TaqMan™ probe-based assay and measured using 7,500 Fast Real-Time PCR System (Applied Biosystems) and the 7,500 v.2.3 software. Expression of each gene transcript was determined relative to the reference gene transcript (*GAPDH*) and normalized to the expression of the target gene using 2^−ΔΔCT^ method. Gene specific primers of *ABCC4* (Assay ID: Mm01226372_m1), *ABCC1* (Assay ID: Mm00456156_m1), *ABCC2* (Assay ID: Mm00496899_m1), *ABCC5* (Assay ID: Mm01343626_m1), *GAPDH* (Assay ID: Mm99999915_g1), *PPARγ* (Assay ID: Mm01184321_m1), *LPL* (Assay ID: Mm00434764_m1), *FABP4* (Assay ID: Mm00445878_m1), *C/EBPα* (Assay ID: Mm07294206_s1), *PGC-1α* (Assay ID: Mm01208835_m1), *ATGL* (Assay ID: Mm00503040_m1), *GLUT4* (Assay ID: Mm00436615_m1) and *CD-36* (Assay ID: Mm00432403_m1) were all procured from Thermo Fisher Scientific. Data were compared with the control group treated with Silencer™ Select Negative Control No. 1 siRNA.

### 2.3 siRNA transfection

Gene silencing of *ABCC4*, *ABCC1*, and *ABCC5* was carried out by siRNA procured from Thermo Fisher Scientific. 3T3-L1 cells were transfected with Silencer™ Select Negative Control No. 1 siRNA, *ABCC4* Silencer™ Select (Assay ID: s108961), *ABCC1* Silencer™ Select (Assay ID: s69747) and *ABCC5* Silencer™ Select (Assay ID: s203177) using TransIT-TKO (MirusBio, Madison, WI), a non-liposomal-based polymeric transfection reagent. TransIT-TKO was diluted with Reduced Serum Medium OPTI-MEM (Gibco). The sequence of si*ABCC4* is sense, 5′- CGAAUGGAAAUAUAACGGAtt-3′, guide strand 5′- UCCGUUAUAUUUCCAUUCGca-3′, siABCC*1* sense 5′-GGCUUAACACCAUAAUGGAtt-3′, guide strand 5′-UCCAUUAUGGUGUUAAGCCga-3′ and si*ABCC5* sense 5′- CCCGAGUGGUUCACAAGAAtt-3′, guide strand 5′-UUCUUGUGAACCACUGGGGcc-3’. For single treatment, 3T3-L cells were seeded at a density of 2 × 10^5^ per well and were treated with 50 nM siRNA on day 6 of the differentiation process. After 48 h, the transfected cells were analyzed by RT-qPCR. For multiple siRNA treatments, cells were treated with 25 nM siRNA on day 0, day 3, and day 6 during the differentiation process, and the cells were analyzed 48 h after the last treatment.

### 2.4 Western blotting analysis

2 × 10^5^ 3T3-L1 cells were seeded per well in 12 well plates and differentiated into mature adipocytes. Total protein was isolated from mature cells using RIPA buffer and was quantified using Pierce™ BCA Protein Assay kit. Isolated proteins (30 µg/well) were electrophoretically resolved using 8%–10% polyacrylamide gels which were then transferred onto the PVDF membrane. Immunochemical detection of protein was performed using *MRP4* (M4I-10, 1:500) antibody (Abcam, Cambridge, MA), and *GAPDH* (14C10, 1:3000) antibody (Cell Signaling Technology, Danvers, MA). Protein-antibody complexes were detected using peroxidase-conjugated affinipure Goat Anti-Rat IgG (H + L) secondary antibody. Protein bands were visualized by Chemidoc (Bio-Rad) using luminol: peroxide (1:1) solution. Data were analyzed using ImageJ software.

### 2.5 Lipid content assays

Lipid accumulation and adipogenesis of 3T3-L1 cells were determined using Oil Red O (ORO) staining. 2 × 10^5^ cells were seeded and differentiated into mature adipocytes. On the day of staining (day 8), cell culture media was removed, and cells were washed with PBS twice. After washing, cells were fixed with 10% neutral buffered formalin for 15 min. Cells were further washed with water and air-dried for 2–3 min. Post drying, 60% isopropyl alcohol was added in each well for 5 min and then cells were stained with 60% ORO working solution for 30 min. The staining reagent was removed from the wells and cells were washed twice with water. Stained lipids inside the cells were visualized under the microscope. Quantification of ORO accumulation in the cells was carried out by eluting the accumulated dye in 100% isopropyl alcohol. The optical density of untreated and treated cells was measured at 540 nm using a BioTek multi-plate reader.

### 2.6 Determination of intracellular cAMP levels

Intracellular cAMP levels were analyzed using a cAMP ELISA kit (Catalog: 581001, Cayman Chemical, Ann Arbor, MI). 2 × 10^5^ cells were seeded in 12 well plates. After 48 h of the last siRNA treatment, cells were treated with forskolin (10 µM) for 30 min in the serum-free media. After 30 min, the cell supernatant was removed, and the cells were washed with PBS. After washing, cells were incubated for 20 min in 0.1 N HCl. Cell lysate was collected, and intracellular cAMP levels were determined using standard manufacturer protocol.

### 2.7 Determination of extracellular prostaglandin E levels

Extracellular prostaglandin E2 (PGE2) levels in the cell culture media were determined using PGE2 ELISA kit-Monoclonal (Catalog: 514010, Cayman Chemical, Ann Arbor, MI) as per the standard manufacturer’s protocol. 3T3-L1 cells (2 × 10^5^) were seeded in 12 well plates and were differentiated into mature adipocytes. Cell culture media in each well were collected 48 h after the last treatment of siRNA and used for the analysis.

### 2.8 Statistical analysis

Statistical analysis was performed using GraphPad Prism 8 software. One-way ANOVA followed by multiple comparison tests were carried out. For statistical analysis, a 95% confidence interval and *p*-value <0.05 were considered significant.

## 3 Results

### 3.1 3T3-L1 cell differentiation and expression of ATP-binding cassette transporters

To gain insights into the expression profile of the *ABCC4* transporter throughout 3T3-L1 cell differentiation, we conducted assessments of both mRNA and protein levels of *ABCC4* at different stages of the process. Isobutyl methylxanthine (IBMX), dexamethasone, and insulin (MDI) hormone treatment stimulates adipogenesis in 3T3-L1 fibroblasts wherein the long elliptical pre-adipocytes get converted into oval-shaped cells associated with lipid droplets in the cytoplasm. We noted that adding an MDI cocktail in the media after 48 h post-confluency of pre-adipocytes showed optimal differentiation with significant lipid deposition compared to differentiation when initiated at pre-confluency (Supplementary Figure S1).

**FIGURE 1:**
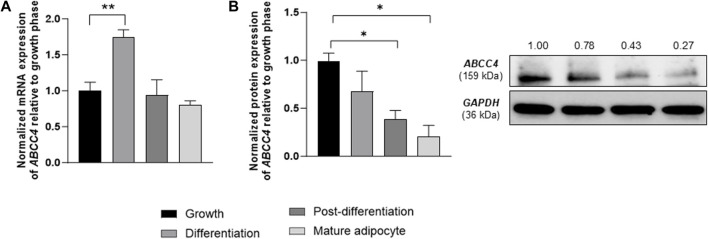
3T3-L1 cell differentiation and expression of ATP-binding cassette transporters. **(A)** *ABCC4* gene expression levels in 3T3-L1 cells at indicated phases of differentiation. Gene expression levels were normalized with *GAPDH* as control. Data are presented as mean + SD (n = 3). **(B)** *ABCC4* protein levels in 3T3-L1 cells at indicated phases of differentiation. Protein levels were normalized relative to *GAPDH* as control. Data are presented as mean + SD (n = 3). One-way ANOVA followed by the Dunnett test was performed. Asterisks represent significant *p* values. *p ≤ 0.05 and **p ≤ 0.01 were considered statistically significant.

Next, post-addition of the MDI cocktail, cells showed a 1.74-fold high expression of the *ABCC4* gene compared to the growth phase (in pre-adipocytes) and a gradual decrease in *ABCC4* expression as cells matured into adipocytes (on day 8). The protein levels of *ABCC4* were much higher (0.8-fold) in pre-adipocytes than in mature adipocytes (Figures 1A, B).

### 3.2 *ABCC4* gene silencing by siRNA

Following confirmation of *ABCC4* expression in 3T3-L1 cells across various differentiation stages, we employed siRNA to investigate *ABCC4* transporter function on 3T3-L1 adipocyte physiology. Initially, siRNA treatment on day 6 of differentiation on adherent 3T3-L1 cells (forward transfection) did not result in *ABCC4* downregulation. However, siRNA treatment by reverse transfection on day 6 reduced *ABCC4* gene expression by 50%, though no significant change in protein expression was observed. Furthermore, reverse transfection altered cell morphology and led to a notable reduction in entrapped lipids (Data not shown).

We employed the non-liposomal lipid/polymeric TransIT-TKO (MirusBio, Madison, WI) to enhance silencing efficiency as a transfection agent. Treatment with siRNA on day 6 of 3T3-L1 cell differentiation resulted in a 0.34-fold change in *ABCC4* gene expression. Interestingly, this silencing effect did not induce changes in cell morphology, and *ABCC4* protein expression levels were unchanged (Supplementary Figure S2). Furthermore, the knockdown of *ABCC4* did not demonstrate any observable effects on downstream targets associated with adipogenesis and lipogenesis (Data not shown).

**FIGURE 2:**
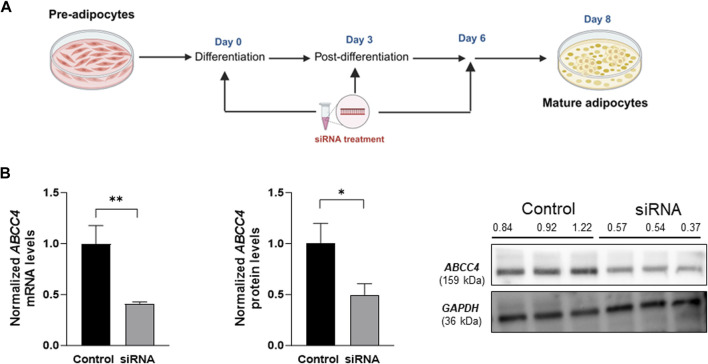
*ABCC4* gene silencing by siRNA. **(A)** Workflow for multiple siRNA treatments at 25 nM each during 3T3-L1 cell differentiation. **(B)** Normalized *ABCC4* gene and protein expression levels post multiple siRNA treatment. Both gene and protein level expression data sets were normalized against *GAPDH* as control. Data are presented as mean + SD (n = 3). An unpaired *t*-test was performed. Asterisks represent significant *p* values. *p ≤ 0.05 and **p ≤ 0.01 were considered statistically significant.

Subsequently, we conducted multiple siRNA treatments on days 0, 3, and 6 during differentiation (Figure 2A). These multiple treatments significantly impacted gene and protein expression levels around 48 h after the last siRNA treatment. We observed approximately a 0.41-fold reduction in *ABCC4* gene expression and a 0.49-fold change in protein levels (Figure 2B).

### 3.3 Crosstalk between ATP-binding cassette transporters in 3T3-L1 mature adipocytes

**FIGURE 3:**
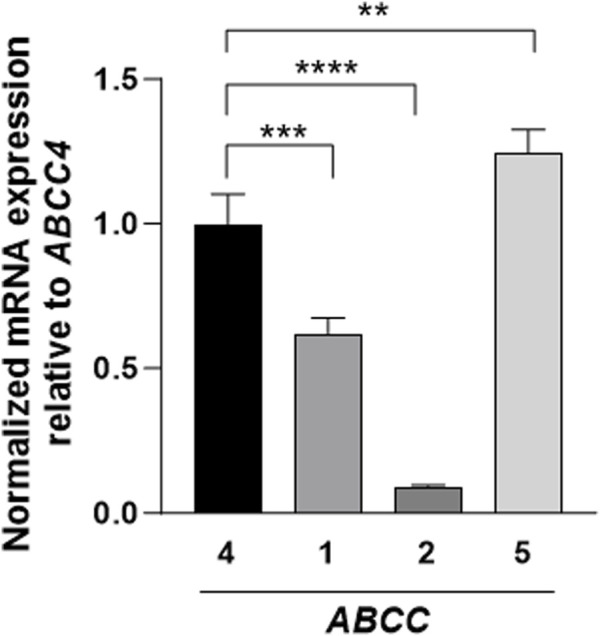
Basal expression of *ABCC* transporters in mature 3T3-L1 cells. Basal gene expression levels of *ABCC* transporters in comparison with *ABCC4*. Gene expressions were normalized against *GAPDH* as control. Data are presented as mean + SD (n = 3). One-way ANOVA followed by the Dunnett test was performed. Asterisks represent significant *p* values. **p ≤ 0.01, ***p ≤ 0.001 and ****p ≤ 0.0001 were considered statistically significant.

**FIGURE 4:**
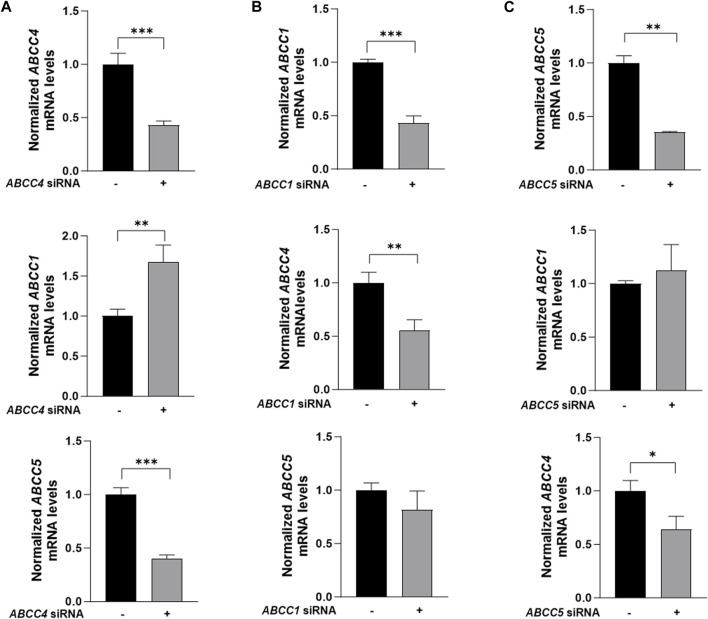
Silencing effect of *ABCC* transporters in 3T3-L1 mature adipocytes. **(A)** Effect of *ABCC4* silencing on *ABCC1* and *ABCC5*. **(B)** Effect of *ABCC1* silencing on *ABCC4* and *ABCC5*. **(C)** Effect of *ABCC5* silencing on *ABCC1* and *ABCC4*. Gene expression levels were normalized with *GAPDH* as control. Data are presented as mean + SD (n = 3). An unpaired *t*-test was performed. Asterisks represent significant *p* values. *p ≤ 0.05, ** *p* ≤ 0.01 and ***p ≤ 0.001 were considered statistically significant.

Among the ATP-binding cassette transporter family ‘C' genes, *ABCC4*, *ABCC1*, *ABCC2*, and *ABCC5* were detected in 3T3-L1 mature adipocytes. Notably, *ABCC2* levels were the lowest amongst other transporters and *ABCC5* expression was found to be 1.24-fold higher as compared to *ABCC4* (Figure 3). Through siRNA-mediated knockdown of *ABCC4* gene function, significant alterations in the gene expression patterns of *ABCC1* and *ABCC5* were observed. Specifically, *ABCC1* expression increased by 1.67-fold, while *ABCC5* expression decreased by 0.60-fold, indicating potential crosstalk among *ABCC* transporters in mature adipocytes (Figure 4A).

To further investigate this crosstalk, individual gene knockdown experiments were conducted, and their effects on other transporters were evaluated. *ABCC1* gene knockdown using siRNA resulted in a 0.45-fold reduction in *ABCC4* expression, while no significant effect was observed on *ABCC5* levels (Figure 4B). Conversely, *ABCC5* siRNA knockdown led to a 0.36-fold change in *ABCC4* gene levels, without impacting *ABCC1* expression (Figure 4C).

Given the high expression levels of *ABCC4* and *ABCC5* in mature adipocytes, we focused on these transporters to investigate crosstalk in 3T3-L1 mature cells.

### 3.4 *ABCC4* and *ABCC5* knockdown promotes lipid recruitment

**FIGURE 5:**
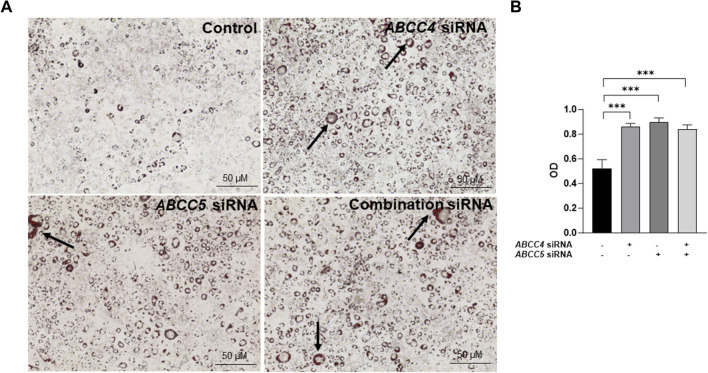
*ABCC4* and *ABCC5* knockdown promotes lipid recruitment. **(A)** Brightfield images of Oil red O staining of 3T3-L1 cells post silencing (Images taken at ×100 magnification). The arrow indicates the lipid droplets. The scale bar represents 50 μM. **(B)** Data represent lipid droplets’ optical density (OD) values following Oil red O staining. Data are presented as mean + SD (n = 3). One-way ANOVA followed by the Dunnett test was performed. Asterisks represent significant *p* values. ***p ≤ 0.001 was considered statistically significant.

Silencing *ABCC4* function in 3T3-L1 cells resulted in a reduction in the expression level of the *ABCC5* gene, and interestingly, we observed that this effect was bidirectional. However, their combined effect did not demonstrate synergy when *ABCC4* and *ABCC5* were simultaneously knocked down. This was evident from the lipid recruitment in 3T3-L1 cells post-maturation. Upon staining with Oil Red O, fully differentiated 3T3-L1 cells treated with siRNA exhibited significantly higher lipid accumulation than those treated with a control (Figure 5A). Upon quantification of lipid accumulation, we noticed that there was no significant difference between the lipid accumulation levels observed in individual gene knockdown *versus* the combination treatment (Figure 5B).

### 3.5 *ABCC4* and *ABCC5* knockdown promotes adipogenesis in 3T3-L1 differentiated cells

**FIGURE 6:**
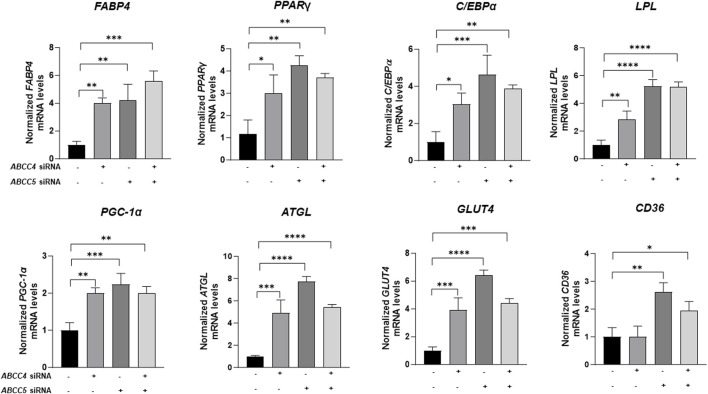
Effect of *ABCC4* and *ABCC5* siRNA knockdown on genes associated with adipogenesis. *PPARγ*-Peroxisome proliferator-activated receptor gamma, *LPL*-Lipoprotein lipase *FABP4*-Fatty acid binding protein 4, *C/EBPα*-CCAAT/enhancer-binding protein-alpha, *PGC-1α*-Peroxisome proliferator-activated receptor-gamma coactivator 1 alpha, *ATGL*- Adipose triglyceride lipase, *GLUT4*- Glucose transporter type 4, *CD36* ^−^ Cluster of differentiation 36. Effect *ABCC* silencing on adipogenic genes. Gene expression levels were normalized with *GAPDH* as control. Data are presented as mean + SD (n = 3). One-way ANOVA followed by the Dunnett test was performed. Asterisks represent significant *p* values. *p ≤ 0.05, **p ≤ 0.01, ***p ≤ 0.001 and ****p ≤ 0.0001 were considered as statistically significant.

After evaluating lipid recruitment following *ABCC4* and *ABCC5* knockdown, we investigated whether *ABCC4/ABCC5* silencing alters adipose physiology by assessing the gene expression of adipogenic markers. Gene expression analysis in completely differentiated cells showed that knocking down *ABCC4* and *ABCC5* function increased the expression of adipogenic genes such as peroxisome proliferator-activated receptor gamma (*PPARγ*), CCAAT/enhancer-binding protein-alpha (*C/EBPα*), fatty acid binding protein 4 (*FABP4*), lipoprotein lipase (*LPL*) and glucose transporter type 4 (*GLUT4*) (Figure 6). *ABCC4* and *ABCC5* knockdown significantly increased *FABP4* expression by 3.99 and 4.19-fold, respectively. For *FABP4* expression, combined knockdown of *ABCC4* and *ABCC5* showed a more significant effect, with approximately a 5.5-fold upregulation compared to the control. Likewise, *PPARγ*, a transcription factor involved in regulating *FABP4* expression and adipogenesis, was upregulated by 2.99-fold following *ABCC4* silencing and by 4.26 after *ABCC5* individual treatment, compared to the control. However, *PPARγ* did not show a synergistic effect with the combination treatment, though the impact of *ABCC5* knockdown was more pronounced than that of *ABCC4* knockdown. *C/EBPα*, reported to be involved in the regulation of *PPARγ* expression and considered an early regulator of adipogenesis (Guo et al., 2015), was found to be upregulated significantly more by *ABCC5* knockdown more considerably than by *ABCC4* siRNA or the combined knockdown (3.88-fold). *LPL* plays a critical role in lipid metabolism by breaking down triglyceride into free fatty acids, which adipose tissue can take up. *ABCC4* and *ABCC5* silencing upregulated *LPL* expression by 2.8 and 5.2-fold respectively. A combination of *ABCC4* and *ABCC5* silencing showed a 5.1-fold increased expression of *LPL*.

*PGC-1α* is a transcription co-activator that interacts with a broad range of transcription factors involved in fatty acid metabolism (Liang and Ward, 2006). Increased expression of *PGC-1α* promotes transcriptional activation of *PPARγ*. siRNA silencing of *ABCC4* and *ABCC5* upregulated the expression of *PGC-1α* by 2 and 2.23-fold, respectively. The combination treatment also upregulated the expression of *PGC-1α,* but at a lesser magnitude than *ABCC5*. *ATGL*, an enzyme that catalyzes the first reaction of lipolysis and releases free fatty acids from triglyceride (Cerk et al., 2018) was also found to be upregulated in siRNA treatment groups. *ABCC5* silencing had a prominent effect on *ATGL* levels compared to *ABCC4* silencing and combined siRNA knockdown. Increased expression of *GLUT4* is an indicator of impaired glucose uptake and energy expenditure that leads to adipose cell hypertrophy due to excessive lipid storage (Wang et al., 2020). Significant upregulation of *GLUT4* after *ABCC4* and *ABCC5* siRNA treatment indicates its role in promoting adipogenesis. *ABCC5* siRNA treatment showed a 6.40-fold increased expression of *GLUT4*, significantly higher than *ABCC4* siRNA and siRNA combination treatment. In addition, the gene involved in fatty acid translocation, *CD36*, is also upregulated at the highest level after *ABCC5* siRNA treatment compared to *ABCC4* and combination. *ABCC4* siRNA treatment did not change the *CD36* gene as compared to negative control siRNA treatment.

### 3.6 *ABCC4* and *ABCC5* mediate the transport of cAMP and prostaglandins (PGE2) in differentiated 3T3-L1 cells

**FIGURE 7:**
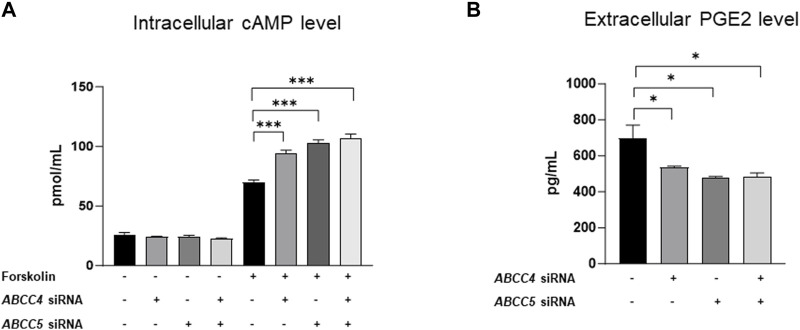
*ABCC4* and *ABCC5* silencing promotes adipogenesis in 3T3-L1 differentiated cells. The deficiency of *ABCC4* and *ABCC5* function alters cAMP and PGE2 levels in intra- and extracellular compartments, respectively. **(A)** Intracellular cAMP levels in 3T3-L1 cells treated with negative control or forskolin or *ABCC4* and *ABCC5* siRNA. **(B)** Intracellular PGE2 levels in 3T3-L1 cells treated with negative control or *ABCC4* and *ABCC5* siRNA. Data are presented as mean + SD (n = 3). One-way ANOVA followed by the Bonferroni (for cAMP) and Dunnett (for PGE2) were performed. Asterisks represent significant *p* values. *p ≤ 0.05 and ***p ≤ 0.001 were considered as statistically significant.

Cyclic nucleotides, bile acids, and prostaglandins are important signaling molecules transported by *ABCC4* and *ABCC5* (Russel et al., 2008). Specifically, cAMP and prostaglandins are recognized as substrates of both *ABCC4* and *ABCC5*, contributing significantly to regulating adipogenesis (Petersen et al., 2008; Fujimori, 2012) Knockdown of *ABCC4* and *ABCC5* led to increased intracellular cAMP levels (Figure 7A), underscoring their role in promoting adipogenesis. Interestingly, simultaneous knockdown resulted in a slightly more pronounced increase in cAMP levels, although the difference was not statistically significant compared to individual treatments. Additionally, we assessed prostaglandin E (PGE2), a known inhibitor of adipogenesis (Coetzee et al., 2005) and observed decreased levels of PGE2 in the extracellular media (Figure 7B). This finding further confirms the role of *ABCC4* and *ABCC5* in promoting adipogenesis in differentiated 3T3-L1 cells.

## 4 Discussion

Adipogenesis is a multi-step process regulated by complex signaling steps, resulting in significant changes in cell morphology (Jakab et al., 2021). After adipogenesis, pre-adipocytes are occupied by large lipid droplets and considered mature adipocytes, which play a crucial role in the regulation of nutrient metabolism (Wang et al., 2013). Past few studies provide evidence of the involvement of transporter protein in regulating nutrient balance. *ABCC4* protein has gained a lot of interest for its role in adipogenesis. Employing a loss-of-function approach, we investigated the specific impact of *ABCC4* on lipid homeostasis. Mice embryonic fibroblast, 3T3-L1 cells were used in this study, which can be considered an ideal model for studying adipogenesis (Ruiz-Ojeda et al., 2016).

Gene silencing in mature adipocytes is associated with challenges that arise due to lipids in the cells, particularly in fully differentiated 3T3-L1 cells. Previous reports also highlighted the hindrance posed by intracellular neutral lipid content in mature 3T3-L1 cells for the entry of positively charged lipid-based transfection reagents (Kilroy et al., 2009). Our approach of utilizing a polymeric non-liposomal transfection reagent successfully overcame this difficulty, ensuring effective silencing of *ABCC4* in differentiated adipocytes. Moreover, the timing of siRNA treatment during the differentiation process significantly influences transfection efficiency and downstream effects on adipogenesis-related targets. Our experiments demonstrated that *ABCC4* silencing on day 6 of differentiation achieved robust knockdown of the *ABCC4* gene. However, the lack of significant impact on adipogenesis suggests that by day 6, the cells are already extensively differentiated into adipocytes, exhibiting minimal morphological changes in response to gene silencing. Multiple siRNA treatments on day 0, day 3, and day 6 showed a silencing effect at both gene and protein levels and affected downstream genes related to adipogenesis.

Various studies have highlighted the outcome of altered expression of *ABCC* transporters. Reports have highlighted failure in the treatment of pediatric patients with acute lymphoblastic leukemia due to altered expression of the *ABCC* transporter gene. Their finding revealed that expression of *ABCC2-6* was elevated and *ABCC1* and *10* were downregulated which is associated with the progression of acute lymphoblastic leukemia (Mehrvar et al., 2019).

Our observations also indicate that silencing the function of *ABCC4* triggers alterations in the levels of other *ABCC* family transporters, suggesting the presence of positive feedback regulation among these transporters. Particularly important was the identified crosstalk between *ABCC4* and *ABCC5*, wherein silencing *ABCC4* led to a subsequent reduction in *ABCC5* expression. This phenomenon underscores the interconnected nature of transporter networks and implies potential regulatory interactions between *ABCC4* and *ABCC5* in adipocyte biology. In addition, another reason behind *ABCC4* and *ABCC5* mutual regulation is shared signaling pathways or common transcription factors. *ABCC4* and *ABCC5* are also involved in nucleotide transport and drug resistance and are reported to be co-regulated by stress response mechanisms (Ritter et al., 2005). *ABCC1* silencing did not show any effect on *ABCC5*, which could be due to the higher expression level of *ABCC5* compared to *ABCC1* in adipocytes, which diminishes any observable regulatory impact of *ABCC1* on *ABCC5*.

The present study also provides proof of concept, demonstrating that silencing *ABCC4* and *ABCC5* disrupts the transport of cAMP and PEG2, pivotal players in the process of adipogenesis (Donepudi et al., 2021). Specifically, inhibition of *ABCC4* function leads to an increase in intracellular cAMP levels. Elevated cAMP levels trigger various downstream signaling pathways crucial for promoting adipogenesis, including upregulation of *C/EBPα* and *PPARγ* expression (Fox et al., 2006). At the basal level, it is difficult to measure the levels of cAMP due to its low abundance and high phosphodiesterase activity in mature adipocytes. Forskolin challenge helps in amplifying the cAMP levels by directly stimulating adenylate cyclase, bringing them to a detectable range (Alasbahi and Melzig, 2012; Cero et al., 2023). The transcriptional activity of *PPARγ* is regulated synergistically by cAMP, which is an important factor for adipogenesis and glucose metabolism (Petersen et al., 2008). In our experiments with *ABCC4* and *ABCC5* knockdown of 3T3-L1 cells, we observed an increase in the expression of *C/EBPα* and *PPARγ,* further supporting this pathway’s role in adipogenesis.

*ABCC4*’s role in transporting prostaglandin E metabolites is also significant in adipogenesis. Research has shown that prostaglandin E2 (PGE2) downregulates the expression of *PPARγ*, a pivotal regulator of adipogenesis (Fujimori, 2012). This anti-adipogenic effect of PGE2 is mediated through activating its cell surface receptors, E-type prostanoid receptors (EP3 and EP4) (Gu et al., 2016). Inhibition of *ABCC4* function reduces the levels of PGE2 in the extracellular space, potentially leading to decreased activation of EP3 and EP4 receptors. Consequently, this reduction in receptor activation could promote adipogenesis by alleviating the anti-adipogenic effects mediated by PGE2. Interestingly, our study also revealed that *ABCC5* influences PGE2 transport, contributing to the upregulation of adipogenesis. The resulting changes in cAMP and PGE2 levels collectively promote adipogenesis in *ABCC4* and *ABCC5* silenced cells.

In conclusion, our study highlights the intricate interplay between *ABCC* transporters and their impact on adipocyte biology. Understanding the functional roles of specific transporters such as *ABCC4* and *ABCC5* enhances our knowledge of adipogenesis regulation and holds promise for potential therapeutic interventions targeting adipocyte function and metabolism.

## 5 Future direction

Considering the findings from our present study, our future investigations aim to explore the response of 3T3-L1 cells under *ABCC4* overexpression conditions and its impact on the degree of adipogenesis. Additionally, during the differentiation phase, we observed an increase in *ABCC4* mRNA levels, which returned to the same level as preadipocyte levels by the time the cells fully matured. However, at the protein level, *ABCC4* expression showed a gradual reduction. This discrepancy could be attributed to the highly differentiating state of 3T3-L1 cells, which may lead to post-translational modifications affecting protein stability or degradation. While we did not explore these mechanisms in the current study, this is an important area for future research better to understand the regulation of *ABCC4* protein expression during adipogenesis. We are also interested in the potential crosstalk among *ABCC* family transporters and plan to delve deeper into understanding its underlying mechanisms. Furthermore, differential metabolomics could help identify *ABCC4* transported molecules that play functional roles in adipogenesis, offering insights into the metabolic pathways influenced by *ABCC4* activity.

## Data Availability

The original contributions presented in the study are included in the article/Supplementary Material, further inquiries can be directed to the corresponding author.

## Abbreviations

ABC, ATP-binding cassette transporters; ATGL, Adipose triglyceride lipase; C/EBP*α*, CCAAT/enhancer-binding protein alpha; cAMP, Cyclic adenosine monophosphate; CD36, Cluster of differentiation 36; EP, E-type prostanoid receptors; FABP4, Fatty acid binding protein 4; GLUT4, Glucose transporter type 4; IBMX, Isobutyl methylxanthine; LPL, Lipoprotein lipase; MDI, Isobutyl methylxanthine, dexamethasone, and insulin; PGC-1α, Peroxisome proliferator-activated receptor-gamma coactivator 1 alpha; PGE2, prostaglandin E2; PPARγ, Peroxisome proliferator-activated receptor gamma.
