# Supplementary material for: Investigating the crosstalk between ABCC4 and ABCC5 in 3T3-L1 adipocyte differentiation
Source: Front Mol Biosci. 2024 Dec 9;11:1498946. doi: 10.3389/fmolb.2024.1498946 (PMC11663720; doi:10.3389/fmolb.2024.1498946)
Supplement: Supplementary file 1 [file Image1.pdf]

Supplementary Figures:

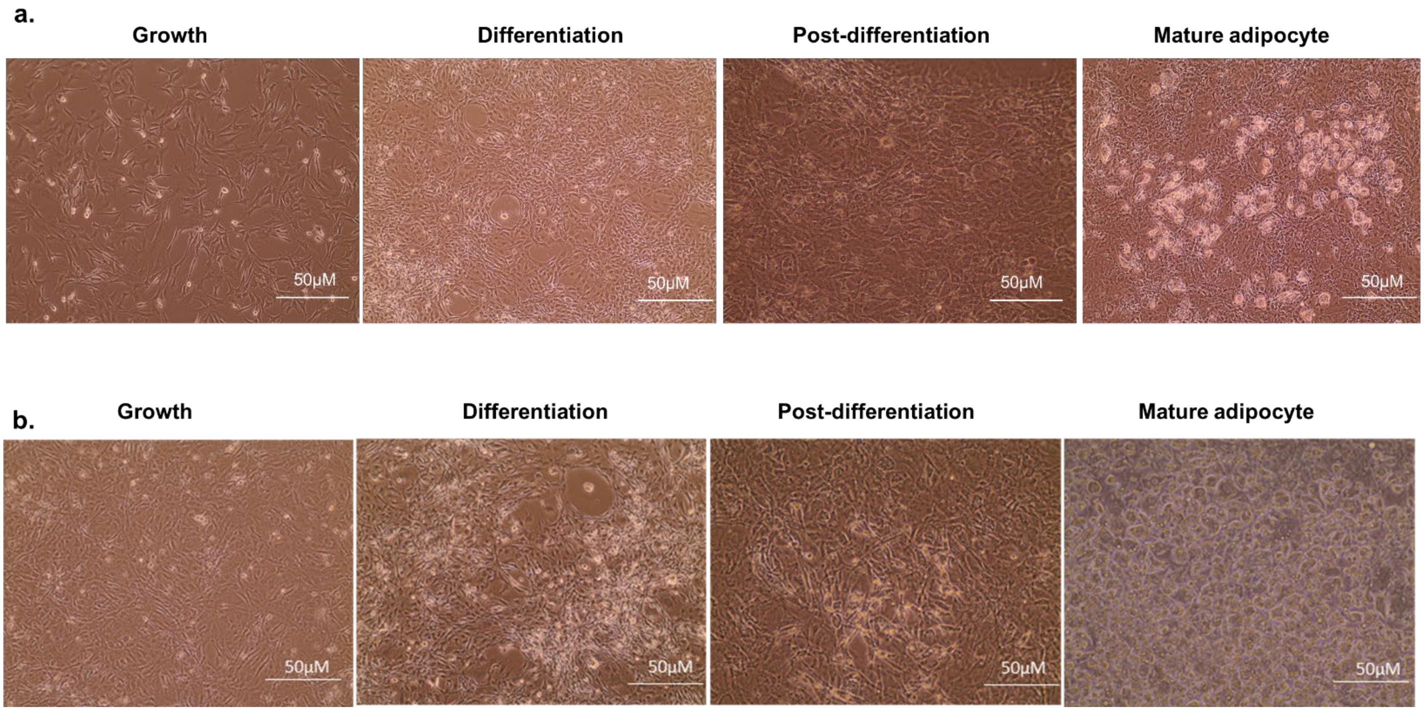

Supplementary Figure 1: Differentiation of 3T3-L1 cell into mature adipocyte. a. Differentiation initiated at pre-confluency. b. Differentiation initiated 48 h post-confluency.

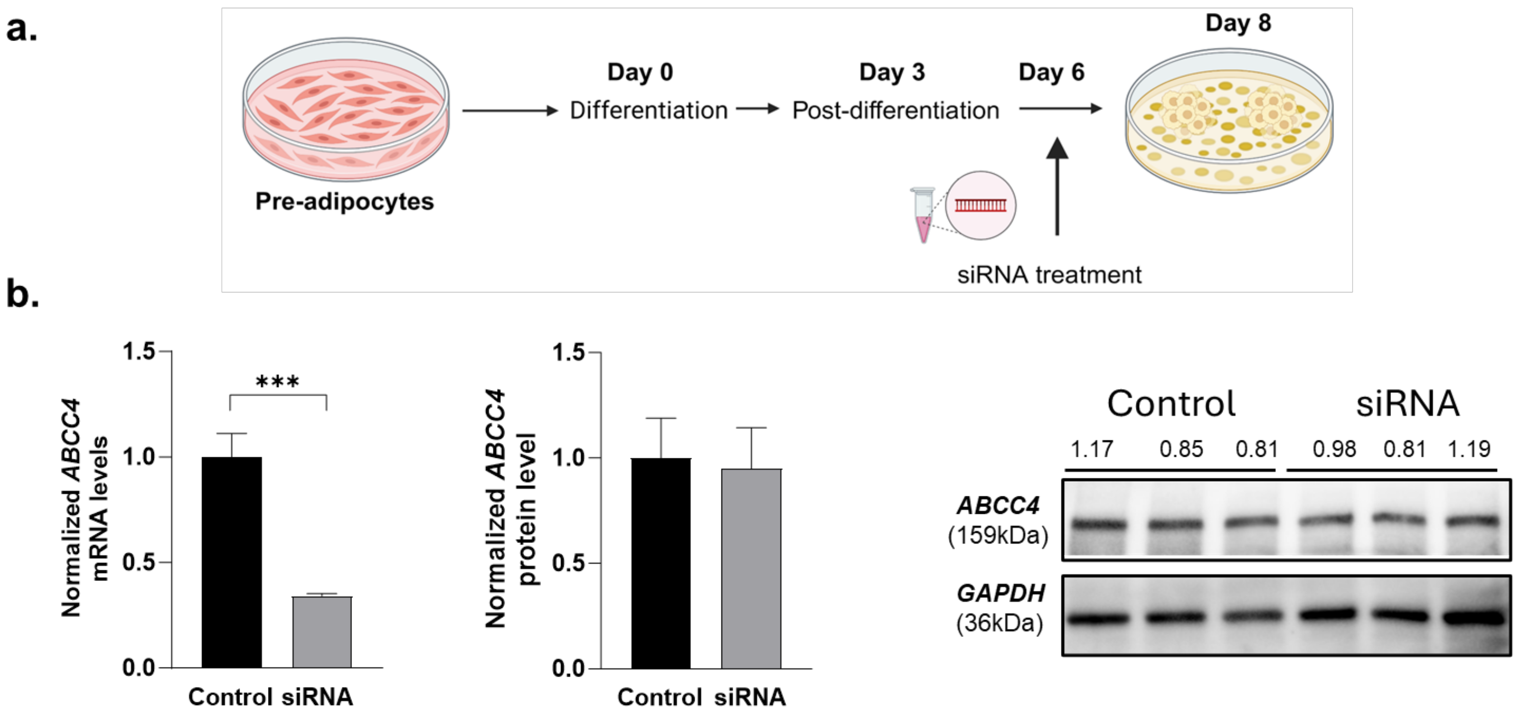

Supplementary Figure 2: *ABCC4* gene silencing by siRNA (Single treatment). a. Workflow for single siRNA treatments at 50 nM each during 3T3-L1 cell differentiation. **b.** Normalized *ABCC4* gene and protein expression levels post single siRNA treatment. Both gene and protein level expression data sets were normalized against *GAPDH* as control. Data are presented as mean+SEM (n=3). An unpaired t-test was performed. Asterisks represent significant p values. ***$p \leq 0.001$ was considered statistically significant.
